# Long-term survival with good functional capacity in unoperated complex congenital heart defect with balanced systemic and pulmonary circulation

**DOI:** 10.1007/s10554-014-0404-1

**Published:** 2014-03-29

**Authors:** Maciej Haberka, Ewa Jastrzębska-Maj, Małgorzata Biedroń, Leszek Szymański, Jolanta Zuziak-Pruska, Zbigniew Gąsior

**Affiliations:** 1 2nd Department of Cardiology, Medical University of Silesia, Ziołowa 45/47, 40-635 Katowice, Poland; 2 Department of Diagnostic Imaging, Upper Silesian Medical Katowice, Katowice, Poland

**Fig. 1:**
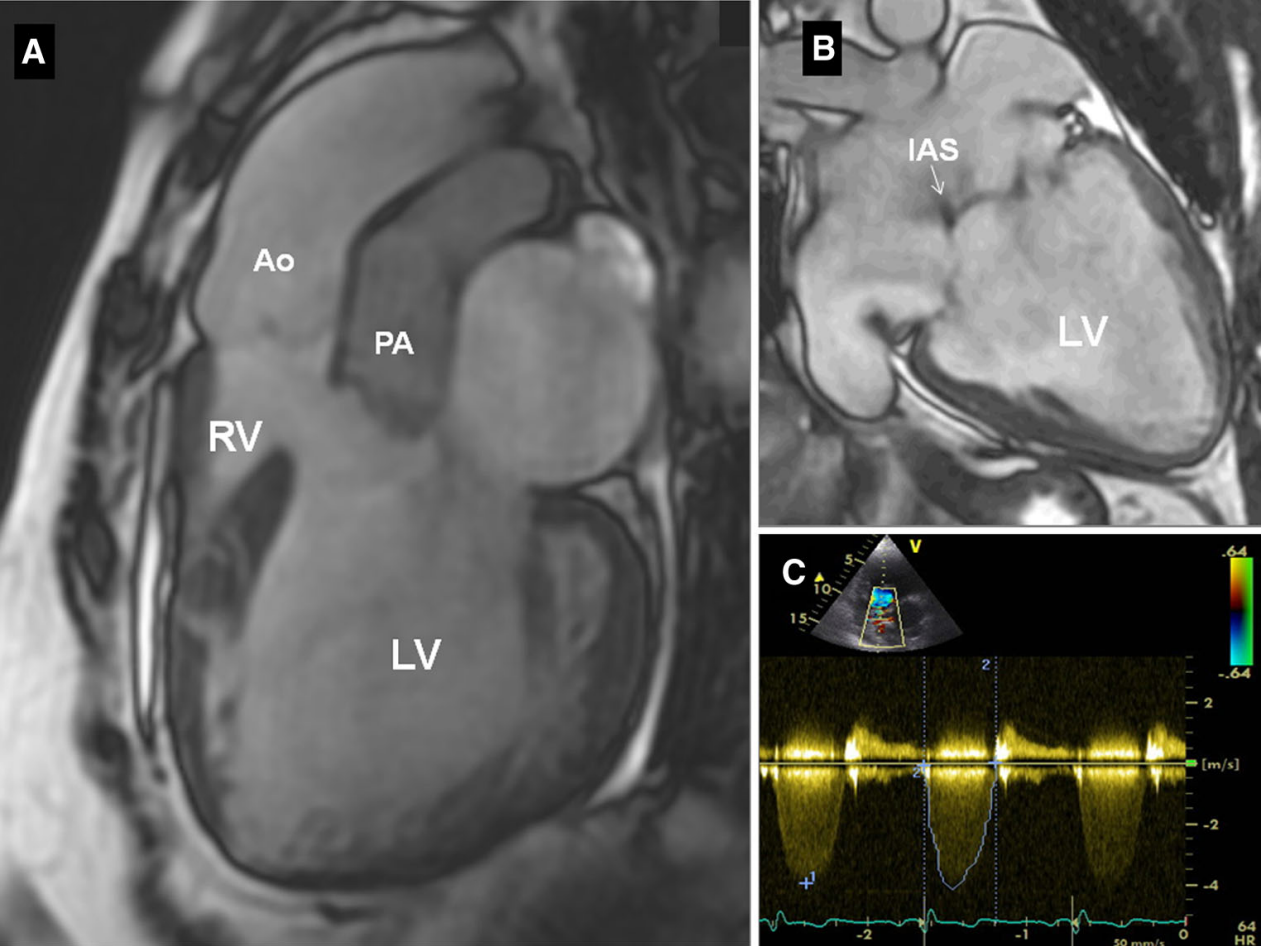
Cardiac magnetic resonance (**a**, **b)** and transthoracic echocardiography (**c)** showing a left-type single ventricle (*LV*), an unrestrictive interventricular septum defect with a rudimentary right ventricle (*RV*), transposition of great arteries (*Ao* aorta;* PA* pulmonary artery), residual interatrial septum (*IAS*) (**b**) and a moderate-to-severe pulmonary stenosis in pulsed wave Doppler (**c**)

A 46-year-old woman was admitted to our Department of Cardiology for stable exertional symptoms of heart failure (NYHA I) and distal mild cyanosis. The medical history revealed a complex congenital heart defect (double-inlet ventricle; DIV) diagnosed early at the infancy, but without palliative surgery or radical repair. The patient was leading a near normal life doing all the house work and working as a full-time physical worker. She had three healthy children delivered through a C-section without cardiovascular complications or significant symptoms progression. None of them was diagnosed with a heart defect. Physical examination revealed a blood pressure of 110/70 mmHg, heart rate 70 bpm, distal mild cyanosis (oxygen saturation 71 %), clubbed fingers, loud systolic heart murmur in the precordial area with a left parasternal thrill and only a mild right heart failure symptoms. Electrocardiogram showed a normal sinus rhythm without atrioventricular conduction disturbances. The major routine laboratory tests abnormalities were: Hb-19.85 g/dl, Ht-61.30 %, Fe-50 mg/dl and NT-proBNP-428 pg/ml. Moreover, a few months earlier she was diagnosed a hypothyroidism and a levothyroxine supplementation was introduced. The performed 6 min walk test was used to assess functional capacity showed a maximal distance of 440 m with a desaturation (75–51 %). Transthoracic echocardiography and cardiac magnetic resonance (CMR) imaging showed a double-inlet left ventricle of normal systolic function with unrestrictive ventricular septal defect and rudimentary right ventricle, d-transposition of great arteries. Both atrio-ventricular valves reveal only a mild regurgitation. There are also concordant atria with normal venous return separated with a residual interatrial septum and no pathology within an aortic arch. The most important for a long survival and balanced systemic and pulmonary circulation is that there is normal flow through the aortic valve without any obstruction and a moderate-to-severe pulmonary stenosis with supravalvular pulmonary stenosis, either (Fig. 1). The usual surgical approach in infants is a palliative shunt operation followed by one of the Fontan operation modifications. DIV is believed to carry a poor prognosis if unoperated with most patients dying before 16 y.o. [1]. Survival into adulthood without any surgical treatment is extremely rare and is found only in patients with a left ventricle-type of single ventricle and similar to the presented case combination of heart defects with normal systolic ventricle function and a moderate-to-severe pulmonary stenosis, which seems a crucial condition of balanced pulmonary circulation. The haemodynamic changes during pregnancy may usually significantly worsen cardiovascular symptoms even long time after a delivery. However, our patient denied significant progression of symptoms and there were no complications during or afterwards which suggest a perfectly balanced circulation. CMR provides most necessary information in the complex congenital heart defect, but a heart catheterization may add unique data on pulmonary artery pressure and pulmonary circulation. However the good functional capacity and only a mild cardiovascular symptoms in our patient encourage us to a strict follow-up for ventricular function, arrhythmia and functional capacity.
